# The prognosis and risk factors of baseline high peritoneal transporters on patients with peritoneal dialysis

**DOI:** 10.1111/jcmm.16819

**Published:** 2021-07-26

**Authors:** Guansen Huang, Yi Wang, Yingfeng Shi, Xiaoyan Ma, Min Tao, Xiujuan Zang, Yinghui Qi, Cheng Qiao, Lin Du, Lili Sheng, Shougang Zhuang, Na Liu

**Affiliations:** ^1^ Department of Nephrology Shanghai East Hospital Tongji University School of Medicine Shanghai China; ^2^ Department of Nephrology Shanghai Songjiang District Central Hospital Shanghai China; ^3^ Department of Nephrology Shanghai Punan Hospital Shanghai China; ^4^ Department of Medicine Rhode Island Hospital and Alpert Medical School Brown University Providence Rhode Island USA

**Keywords:** hyperuricemia, mortality, peritoneal dialysis, peritoneal solute transport rate, technique failure

## Abstract

The relationship between baseline high peritoneal solute transport rate (PSTR) and the prognosis of peritoneal dialysis (PD) patients remains unclear. The present study combined clinical data and basic experiments to investigate the impact of baseline PSTR and the underlying molecular mechanisms. A total of 204 incident CAPD patients from four PD centres in Shanghai between 1 January 2014 and 30 September 2020 were grouped based on a peritoneal equilibration test after the first month of dialysis. Analysed with multivariate Cox and logistic regression models, baseline high PSTR was a significant risk factor for technique failure (AHR 5.70; 95% CI 1.581 to 20.548 *p *= 0.008). Baseline hyperuricemia was an independent predictor of mortality (AHR 1.006 95%CI 1.003 to 1.008, *p *< 0.001) and baseline high PSTR (AOR 1.007; 95%CI 1.003 to 1.012; *p *= 0.020). Since uric acid was closely related to high PSTR and adverse prognosis, the in vitro experiments were performed to explore the underlying mechanisms of which uric acid affected peritoneum. We found hyperuricemia induced epithelial‐to‐mesenchymal transition (EMT) of cultured human peritoneal mesothelial cells by activating TGF‐β1/Smad3 signalling pathway and nuclear transcription factors. Conclusively, high baseline PSTR induced by hyperuricaemia through EMT was an important reason of poor outcomes in CAPD patients.

## 1 INTRODUCTION

Peritoneal dialysis (PD) is now globally used as an effective replacement therapy for patients with end‐stage renal disease (ESRD). Ultrafiltration failure and cardiovascular events are still the main reasons for the mortality and withdrawal of long‐term PD patients.^1^ The success of PD depends on the integrity of the peritoneal structure and function. It has been demonstrated that the peritoneum may exhibit submesothelial thickening and vasculopathy at the time of PD catheter insertion in patients with ESRD.^2^ Previous study had also confirmed that intraperitoneal and systemic inflammation increases during the first year of PD therapy and inflammation may partly be responsible for the development of a high peritoneal solute transport rate (PSTR).^3^

The most common peritoneal functional alteration is impaired ultrafiltration and decreased dialysis efficiency caused by fast PSTR.^4^ Ultrafiltration failure is the main limitation of long‐term PD treatment. Peritoneal transport function refers to the permeability of the peritoneum to transport small solutes. PSTR is measured by dialysate‐to‐plasma (D/P) ratios of low‐molecular‐weight solutes through the peritoneal equilibration test (PET). A high PSTR leads to rapid re‐absorption of glucose and increasing in protein loss, which followed by fluid overload and malnutrition.^5^ Since the transport function of peritoneal membrane varies widely among individuals, it is more accurate to study based on different types of fast transporters. There may exist two distinct types of high transporters, the early inherent phenotypes and the late acquired type.^6^, ^7^, ^8^ The late acquired type, a consequence of continuous exposure to bioincompatible PD solutions, has no longer been considered predictors of poor outcomes with the application of automated peritoneal dialysis (APD) and the icodextrin‐based PD solution.^5^, ^9^, ^10^, ^11^ While among those PD patients with early inherent phenotypes of baseline high PSTR, several previous studies demonstrated that they had more technical failure and higher mortality rate.^12^, ^13^, ^14^ However, there are also studies showed the opposite results.^15^, ^16^, ^17^, ^18^ The discrepancy may due to differences in the study population, regional diversity, sample size, primary disease of chronic kidney disease (CKD) or other chronic illness burden is hard to homogenize. According to these existing research results, we still do not fully understand whether a high baseline PSTR was associated with poor prognosis.

The potential causes of high PSTR might be increased peritoneal capillary perfusion and vascular numbers, both of which may be inherent or acquired. During long‐term PD, peritoneal membrane is continuously exposed to high dextrose concentration dialysate, coexisted with the other inflammatory stimuli such as peritonitis, and the peritoneal membrane may develop many structural abnormalities, including angiogenesis and submesothelial fibrosis.^19^, ^20^ While persistent intraperitoneal inflammation may ultimately lead to fast PSTR and peritoneal fibrosis (PF).^2^, ^21^, ^22^, ^23^ Another study considered that inflammation, along with comorbidity and low serum albumin, was independent predictors of higher baseline peritoneal permeability.^24^ Moreover, Pletinck A. et al. reviewed factors including haemoglobin A1c level, salt intaken and genetic polymorphisms which have important effects on the peritoneal membrane and can result in variability of peritoneal function.^25^ Therefore, there is no consistent view of the cause of the increased peritoneal permeability.

In terms of molecular mechanism, epithelial‐to‐mesenchymal transition (EMT) of peritoneal mesothelial cells has been proved to be associated with high peritoneal transport^26^ which occurred even early in CAPD and will be developed during long‐term exposure to high glucose dialysate, mechanical denudation, profibrotic factors such as TGF‐β and inflammatory cytokines.^27^ When EMT occurs, the mesothelial cells adopt a more fibrogenic characteristic and acquire a proliferative, migratory and invasive phenotype.^28^ As a result, a large amount of neovascularization and accumulation of extracellular matrix accelerate tissue fibrosis, alter peritoneal transport status and lead to ultrafiltration failure.^27^, ^29^ Recently, Mizuiri et al. evaluated the association between PSTR and the expression of effluent markers related to EMT and they found effluent hepatocyte growth factor (HGF), vascular endothelial growth factor (VEGF) and interleukin‐6 (IL‐6) levels were significantly higher in the patients with high transport rate.^26^ Moreover, a review newly published by our team systematically summarizes the angiogenic effect of VEGF. VEGF is associated with high peritoneal transport rate while impaired mesothelial cells are the major sources of VEGF in the peritoneum.^30^

Since the evaluation of peritoneal function of patients is after the start of peritoneal dialysis, there are still a large number of patients with initial high PSTR who undergo PD and their prognosis is not clear. This study was undertaken to evaluate the subsequent overall survival and technique survival of the PD patients grouped by their baseline peritoneal transport status, analyse independent predictive risk factors of high baseline peritoneal transport status and explore its potential pathogenesis through in vitro experiments.

## 2 MATERIALS AND METHODS

### 2.1 Ethics statement

This study was conducted according to the guidelines of the Helsinki Declaration. Participant consents were obtained. Human Research Ethics Committee of Shanghai East Hospital Affiliated to Tongji University School of Medicine, Baoshan Branch of Shanghai First People's Hospital, Shanghai Songjiang District Central Hospital and Shanghai Punan Hospital approved this study (ChiCTR2000030053).

### 2.2 Study design and participants

The present analysis was a multicentre retrospective cohort study that included all of the incident PD patients, aged between 18 and 75 years, started continuous ambulatory peritoneal dialysis (CAPD) between 1 January 2014 and 30 September 2020 in the Department of Nephrology in Shanghai East Hospital Affiliated to Tongji University School of Medicine, Baoshan Branch of Shanghai First People's Hospital, Shanghai Songjiang District Central Hospital and Shanghai Punan Hospital. All patients had indwelled Tenckhoff double‐polyester sheathed PD catheters and initiated PD with 1.5% or 2.5% dextrose PD fluid (Baxter Healthcare, Guangzhou, China) and performed CAPD 3 to 5 times a day, and daily dialysis dose ranged from 6000 to 10000ml. All the participants underwent a PET after the first month of PD treatment. The exclusion criteria were as follows: patients who underwent PD treatment for less than 6 months, aged less than 18 years or over than 75 years, patients who suffered from peritonitis within the first 3 months, patients who had malignant tumour, liver cirrhosis, active tuberculosis, history of acute myocardial infarction and major surgical trauma within 3 months before starting PD, patients who initiated PD in other PD centres and previously accepted haemodialysis (HD) or kidney transplantation. These enrolled patients were followed until cessation of PD, death or on 30 September 2020.

### 2.3 Demographic and Clinical Data

The baseline data collected consist of information about the demographic details, clinic and biochemical tests and a limited range of comorbidities including diabetes mellitus (DM), hypertension and cardiovascular disease (CVD). CVD included previous and present history of congestive heart failure, ischaemic heart disease or cerebrovascular disease. A fasting venous blood sample was collected before the morning exchange. Blood biochemical examination was analysed by standard techniques. Corrected serum calcium (cSCa) was correction with the following formula: cSCa = serum Ca + 0.8*(4.0‐serum albumin [ALB]) (if serum ALB < 4g/dl).

### 2.4 Peritoneal equilibration test

Baseline PET was performed 1 month after initial of dialysis. According to Twardowski,^31^ a standard 4‐h dwell period was used, using a 2.5% dextrose PD solution for a 2 L volume exchange after 2.5% dextrose PD solution dwelling overnight (8 ~ 12 h). Collecting PD effluent sample at 0, 2 and 4 h and venous blood sample at 2 h, respectively. Both dialysate and plasma glucose and creatinine were measured. According to dialysate: plasma creatinine ratio at 4 h (D:P Cr 4 h), peritoneal transport status was categorized as low (L), low average (LA), high average (HA) and high (H) (L<0.50, LA 0.5–0.64, HA 0.65–0.80, H≥0.81).

### 2.5 Outcomes

The endpoint of the study was the patient status (dead or alive) or technical failure like transferring to HD at termination of the follow‐up period (30 September 2020).

### 2.6 Antibodies and reagents

Antibodies to p‐Smad3 (#9520), Smad3 (#9523), E‐cadherin (#3195), Cyclin E (#20808), Snail (#3879) and Vimentin (#3932) were purchased from Cell Signaling Technology. Antibodies to GAPDH (sc‐32233), Collagen I (A2) (sc‐28654), TGFβRI (sc‐399) and PCNA (sc‐71858) were purchased from Santa Cruz Biotechnology. Antibody to Slug (ab27568) and DAPI (ab104139) was purchased from Abcam. The Cell Counting Kit‐8 (CCK‐8) proliferation assay kit was purchased from Beyotime Biotechnology. Antibody to α‐SMA (A2547) and uric acid (U0881) was obtained from Sigma‐Aldrich. Reactive oxygen species (ROS) assay kit was purchased from Nanjing Jiancheng Bioengineering Institute (Nanjing, China). Peritoneal dialysis fluid (2.5%) was purchased from Baxter Healthcare (Guangzhou, China).

### 2.7 Cell culture and treatments

Human peritoneal mesothelial cells (HPMCs) (kind gifts from Haiping Mao at Sun Yat‐Sen University, Guangzhou, PR China) were cultured in Dulbecco's modified Eagle's medium (DMEM) with F12 containing 10% foetal bovine serum (FBS), 1% penicillin and streptomycin in an atmosphere of 5% CO2 and 95% air at 37°C. To determine the effect of uric acid stimulation on EMT and proliferation, HPMCs were starved for 24 h with 0.5% FBS in DMEM/F12 and then exposed to uric acid in different doses (200, 40 and 800 µM) for 36 h before cell harvesting. In addition, time‐dependent manner of uric acid (800 µM) was taken for different exposure times (12, 24 and 36 h). In order to confirm the additive effect of uric acid and high glucose (HG) in HPMCs, uric acid (800 µM) and 2.5% HG peritoneal dialysis fluid were used singly or in combination for 36 h before cell harvesting. All of the in vitro experiments were repeated for at least three times.

### 2.8 CCK‐8 proliferation assay

The CCK‐8 proliferation kit was used according to the manufacturer's instructions. HPMCs were starved for 24 h with DMEM/F12 containing 0.5% FBS and then exposed to uric acid in different doses (200, 400 and 800 µM). After 36 h, the original culture medium was removed, and 100 µl new DMEM/F12 medium containing 10 µl CCK‐8 was added to each well in a 96‐well plate for 37°C incubation for an additional 4 h. The final optical density values were read at 450 nm.

### 2.9 Wound‐healing assay

HPMCs were seeded into a 6‐well plate and allowed to reach 90% confluence. A scratch wound was created on the cell surface using a micropipette tip. Then, cells were washed with PBS in three times and incubated in serum‐free DMEM/F12 with uric acid (800 µM). Photomicrographs (×40 objective magnification) of migrating cells were taken at 0 and 36 h. The width of the wound was measured using ImageJ software (National Institutes of Health, Bethesda, MD, USA). The migratory rate was calculated as (A − B) / A × 100%, where A and B reflect the width of the wound at 0 and 36 h, respectively.

### 2.10 Reactive oxygen species assay

ROS level was examined with a ROS assay kit that sets DCFH‐DA as the probe. HPMCs were grown in 6‐well plates and were incubated in serum‐free media containing DCFH‐DA (10 μM) in the presence of groups for 60 min at 37°C and 5% CO2 in the dark. After washing with PBS for three times, the positive cells of DCFH‐DA were measured using immunofluorescence photography.

### 2.11 Immunoblot analysis

Cell lysates were collected from each group. Immunoblot analysis was conducted as described previously.^32^ The densitometry analysis of immunoblot results was conducted by using ImageJ software (National Institutes of Health, Bethesda, MD, USA).

### 2.12 Immunofluorescence staining

Immunofluorescence staining was carried out according to the procedure described in our previous study.^33^ HPMCs from different treatment groups were immobilized and incubated with primary antibodies against α‐SMA or E‐cadherin, and then Texas Red‐ or FITC‐labelled secondary antibodies (Invitrogen).

### 2.13 Statistical analysis

Results are expressed as mean ± SD for continuous data and as frequencies (*n*) and percentages (%) for categorical data. Data distribution normality was evaluated by the Kolmogorov‐Smirnov test. A comparison among the different peritoneal transport types was performed by analysis of variance (ANOVA, parametric distribution) or the Kruskal‐Wallis test (non‐parametric distribution). The Kaplan‐Meier survival curves were drawn for each event of interest (patient survival and technique survival), and the log‐rank test was used to compare curves. Univariate and multivariate Cox proportional hazards models were used to search significant risk factors associated with study outcomes. Data were censored at the time of renal transplantation; 30 September 2020 or transfer to haemodialysis for the overall survival analyses, whereas the death‐censored technique analyses were censored at the time of renal transplantation; death; or 30 September 2020. Multivariate logistic regression modelling was used for the analysis of risk factors associated with baseline high PSTR. A two‐tailed *p* value <0.05 was considered statistically significant. In multivariate model, the adjustment variables included peritoneal transport category; age; gender; smoking status; BMI category; weekly residual renal Kt/V; the presence or absence of hypertension; CVD and diabetes, the covariate of laboratory parameters included ALB; C‐reactive protein (CRP); cSCa; cardiac troponin (cTnT); haemoglobin (Hb); glycosylated haemoglobin (HbA1c); phosphate (P); parathyroid hormone (PTH); serum creatinine (Scr); total cholesterol (TC); triglyceride (TG); UA; and 4‐h D/P Cr.

All the in vitro experiments were conducted at least three times. Data depicted in graphs represent the means ± SEM for each group. Intergroup comparison was made using one‐way analysis of variance. Multiple means were compared using Tukey's test. The differences between two groups were determined by Student's *t* test. Statistical significant difference between mean values was marked in each graph. *p *< 0.05 was considered significant.

The statistical analyses were conducted by using IBM SPSS Statistics 20.0 (version X; IBM).

## 3 RESULTS

### 3.1 Patient Characteristics

A total of 204 patients with complete data were enrolled, and there was a preponderance of male patients (55.4%). The mean age of the participants was 61.86 ± 10.98 years. Also, chronic diseases such as DM (46.57%), hypertension (71.56% standard control) and CVD (25.98%) were counted. All patients were categorized by their baseline peritoneal transport status, 32 patients (15.69%) were classified as H group, 100 patients (49.02%) as HA group, 54 patients (26.47%) as LA group and 18 patients (8.82%) as L group. DM and hypertension had similar proportion in each group. Except BMI and TG, other clinic characteristics did not show great differences among each group. The main characteristics according to the peritoneal transport status are summarized in Table 1.

**TABLE 1:** Baseline clinical parameters of patients on continuous ambulatory peritoneal dialysis

Parameters	Total Population (*n* = 204)	L (*n* = 18)	LA (*n* = 54)	HA (*n* = 100)	H (32)	*p* value
Age (y)	61.86 ± 10.98	66.00 ± 7.93	62.09 ± 10.56	61.58 ± 11.00	60.03 ± 12.82	0.090
Male *n* (%)	113 (55.4%)	10 (55.5%)	31 (57.4%)	50 (50.0%)	22 (68.76%)	0.315
DM *n* (%)	95 (46.57%)	12 (66.67%)	27 (50.0%)	51 (51.0%)	19 (59.38%)	0.073
Smoking *n* (%)	32 (15.69%)	2 (11.11%)	6 (11.11%)	18 (18.0%)	6 (18.75%)	0.057
CVD *n* (%)	53 (25.98%)	6 (33.33%)	13 (24.07%)	22 (22.0%)	12 (37.50%)	0.307
BP <140/90 mmHg *n* (%)	146 (71.56%)	9 (50.0%)	40 (74.07%)	71 (77.0%)	26 (81.25%)	0.091·
BMI (kg/m^2^)	23.59 ± 3.37	24.12 ± 4.27	23.64 ± 2.96	23.48 ± 3.50	23.56 ± 3.13	0.043
Weekly peritoneal Kt/V	1.51 ± 0.39	1.66 ± 0.40	1.53 ± 0.36	1.54 ± 0.38	1.34 ± 0.46	0.424
Weekly residual renal Kt/V	0.57 ± 0.54	0.57 ± 0.52	0.50 ± 0.47	0.64 ± 0.55	0.51 ± 0.65	0.450
4 h D/P Cr	0.67 ± 0.13	0.43 ± 0.04	0.58 ± 0.04	0.70 ± 0.03	0.85 ± 0.03	<0.001
Hb (g/L)	99.33 ± 20.30	99.61 ± 21.03	98.35 ± 20.69	100.91 ± 20.23	95.88 ± 19.88	0.925
Alb (g/L)	32.17 ± 5.07	31.63 ± 4.20	33.24 ± 5.16	32.40 ± 5.07	29.97 ± 4.90	0.802
UA (μM)	417.00 ± 118.58	386.43 ± 91.75	400.35 ± 87.84	412.33 ± 127.67	482.20 ± 122.67	0.195
Scr (μM)	796.73 ± 320.28	648.56 ± 231.89	816.14 ± 314.14	792.04 ± 341.16	848.98 ± 294.80	0.575
TG (mM)	1.77 ± 1.47	2.26 ± 1.89	1.85 ± 1.33	1.47 ± 0.91	1.52 ± 0.86	0.024
TC (mM)	4.00 ± 1.14	3.84 ± 1.26	4.01 ± 1.37	3.79 ± 0.92	4.02 ± 1.17	0.576
CRP (mg/L)	13.49 ± 28.54	11.02 ± 10.68	16.78 ± 33.68	14.15 ± 31.68	7.26 ± 8.02	0.491
cTnT (ng/L)	0.57 ± 0.77	0.78 ± 1.10	0.72 ± 0.87	0.50 ± 0.72	0.44 ± 0.49	0.266
PTH (ng/L)	226.14 ± 257.71	196.32 ± 176.67	307.27 ± 337.11	177.46 ± 188.79	254.89 ± 290.42	0.168
cSCa (mM)	0.80 ± 0.59	0.82 ± 0.58	0.84 ± 0.55	0.82 ± 0.61	0.65 ± 0.60	0.696
P (mM)	1.55 ± 0.49	1.60 ± 0.43	1.68 ± 0.46	1.48 ± 0.53	1.48 ± 0.45	0.998
HbAlc (%)	5.74 ± 1.40	5.74 ± 1.33	5.72 ± 1.53	5.82 ± 1.39	5.51 ± 1.28	0.652

Note

cSCa = serum Ca + 0.8*(4.0‐serum ALB) (if serum ALB<4 g/dl);

Abbreviations: 4 h D/P Cr, dialysate:plasma creatinine ratio at 4 h; Alb, albumin; BMI, body mass index; Bp, blood pressure; CRP, C‐reactive protein; cSCa, corrected serum calcium, cTnT, cardiac troponin; CVD, cardiovascular disease; DM, diabetes mellitus; H, high; HA, high average; Hb, Haemoglobin; HbA_1_c, glycosylated haemoglobin; L, low; LA, low average; P, phosphate; PTH, parathyroid hormone; Scr, serum creatinine; TC, total cholesterol; TG, triglyceride; UA, uric acid.

### 3.2 Outcomes of CAPD patients

**FIGURE 1:**
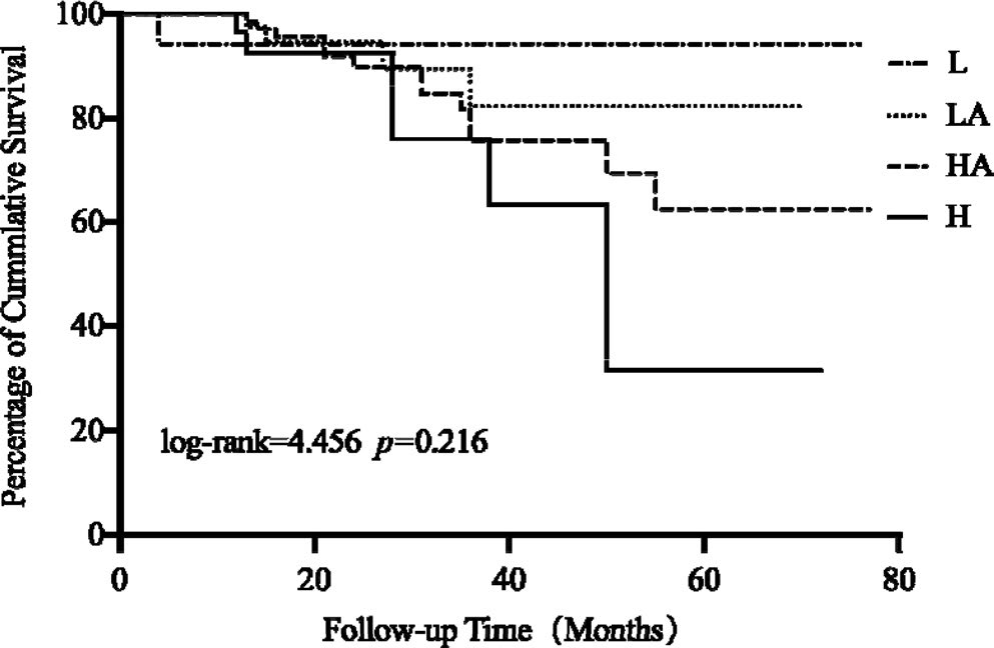
Kaplan‐Meier analysis of overall survival of the four peritoneal transport classes. HA, high average; H, high; L, low; LA, low average

The total average survival time of the study patients was 63.06 ± 2.52 months (95% confidence interval [CI] 58.12 to 67.99), 146(71.57%) patients were alive on PD, and 25 (12.25%) patients had died. There is no significant difference in all‐cause mortality among each group (log‐rank = 4.456 *p *= 0.216) as shown in Figure 1. Baseline peritoneal transport status was not associated with overall survival in Cox proportional hazards model analyses. But it showed strong associations between UA and mortality (adjusted hazard ratio [AHR] 1.006, 95% CI 1.003 to 1.008, *p *< 0.001) when we treated UA as a continuous variable (Table 2). Then, we stratified the participants into 4 groups of SUA levels according to the quartile as follows: ≤341 μM, 342–402, 403–474 and ≥475 μM and found the mortality was increased in the highest quartiles based on Kaplan‐Meier analysis (log‐rank = 16.892 *p *= 0.001), but there was no statistically significant difference in a multivariate Cox proportional hazards model analyses. In addition, total cholesterol (TC) was found to be inversely related to all‐cause mortality (AHR 0.606, 95% CI 0.400 to 0.917, *p *= 0.018).

**TABLE 2:** Risk factors for overall mortality based on the results of univariate and multivariate of Cox proportional hazards analysis

Characteristic (*n* = 204)	Univariate		Multivariate	
	HR (95%CI)	*p*	HR (95%CI)	*p*
D/P Cr 4 h				
L (<0.5)	0. 508 (0.993–2.789)	0. 436		
LA (0.5–0.64)	ref			
HA (0.65–0.80)	1.195 (0.349–4.091)	0. 776		
H (≥0.81)	2.561 (0.823–7.973)	0. 105		
Age (per year)	0.996 (0.960–1.033)	0. 824		
Male Gender	1.121 (0.511–2.461)	0. 776		
BMI				
<18.5 (kg/m^2^)	2.820 (0.366–21.739)	0. 320		
18.5 ~ 23.9 (kg/m^2^)	ref			
24 ~ 27.9 (kg/m^2^)	3.628 (0.373–35.240)	0. 267		
≥28 (kg/m^2^)	2.430 (0.307–19.212)	0. 400		
DM	1.352 (0.607–3.012)	0. 461		
CVD	0.857 (0.341–2.151)	0. 742		
Smoking	1.131 (0.337–3.795)	0. 862		
Bp <140/90 mmHg	0.587 (0.254–1.266)	0. 166		
Hb (per 1 g/L)	0.999 (0.980–1.019)	0. 957		
HbA_1_C (per 1 mM)	0.816 (0.577–1.155)	0. 251		
Alb (per 1 g/L)	0.929 (0.863–1.001)	0. 052		
SCr (per 1 μM)	1.001 (1.000–1.002)	0. 146		
UA (per 1 μM)	1.004 (1.002–1.007)	<0.001	1.006 (1.003–1.008)	<0.001
TG (per 1 mM)	0.794 (0.518–1.218)	0. 291		
TC (per 1 mM)	0.733 (0.511–1.051)	0. 091	0.606 (0.400–0.917)	0.018
cSCa (per 1 mM)	0.831 (0.417–1.654)	0. 598		
P (per 1 mM)	1.290 (0.575–2.656)	0. 507		
PTH (per 1 ng/L)	1.001 (1.000–1.001)	0. 286		
cTnT (per 1 ng/ml)	1.071 (0.650–1.764)	0. 788		
CRP (per 1 mg/L)	1.006 (0.998–1.014)	0.178		
Weekly peritoneal kt/v (per 1 unit)	0.284 (0.085–0.950)	0. 041		
Weekly residual renal kt/v (per 1 unit)	0.715 (0.323–1.585)	0.409		

Note

cSCa = serum Ca + 0.8*(4.0‐serum ALB) [if serum ALB<4g/dl].

Abbreviations: 4 h D/P Cr, dialysate:plasma creatinine ratio at 4 h; Alb, albumin; BMI, body mass index; Bp, blood pressure; CRP, C‐reactive protein; cSCa, corrected serum calcium, cTnT, cardiac troponin; CVD, cardiovascular disease; DM, diabetes mellitus; H, high; HA, high average; Hb, Haemoglobin; HbA_1_c, glycosylated haemoglobin; L, low; LA, low average; P, phosphate; PTH, parathyroid hormone; Scr, serum creatinine; TC, total cholesterol; TG, triglyceride; UA, uric acid.

**FIGURE 2:**
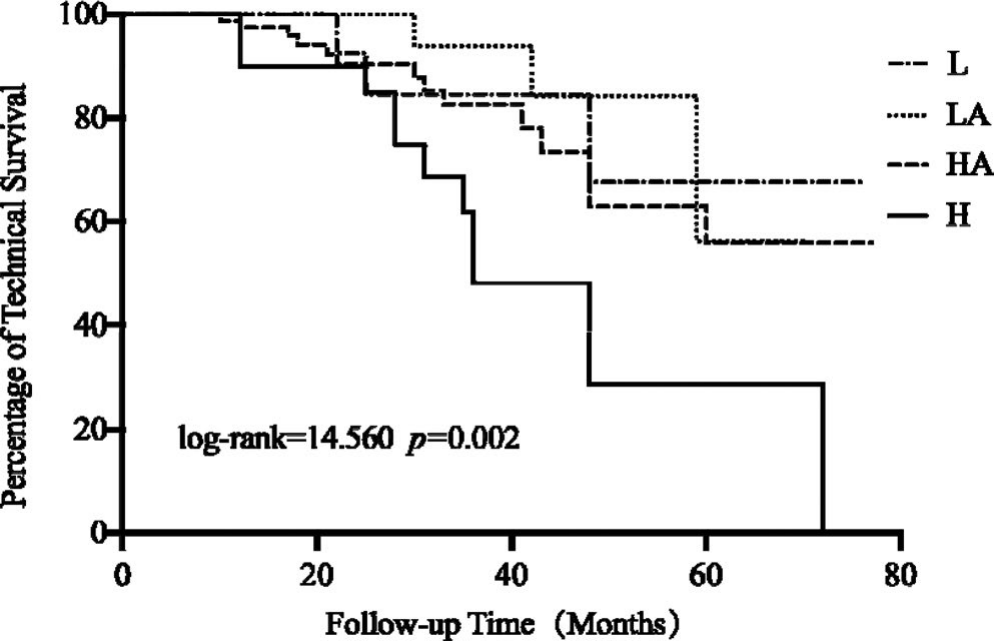
Kaplan‐Meier analysis of death‐censored technique survival of the four peritoneal transport classes. HA, high average; H, high; L, low; LA, low average

The average death‐censored technique survival time for the overall population was 58.96 ± 2.49 m (95% CI 54.09 to 63.83). 33 of 204 CAPD patients transferred to HD. The reasons for technique failure were listed in Table 3. The average technique survival time of the H transporters was 44.51 ± 5.05 m, which was the shortest among the four groups (log‐rank = 14.56, *p *= 0.002) as shown in Figure 2. Taking low average transporters as reference, death‐censored technique survival was significantly decreased in high transporters (AHR2.740, 95% CI 1.280 to 5.866, *p *= 0.009) in univariate Cox proportional hazards model analyses. After multivariate adjusted, high transporter status was still a significant, independent predictor of death‐censored technique failure (AHR 5.70, 95% CI 1.581 to 20.548, *p *= 0.008) (Table 4).

**TABLE 3:** Reasons for technique failure

	L (*n* = 18)	LA (*n* = 54)	HA (*n* = 100)	H (*n* = 32)	Total (204)
Peritonitis	1	1	6	4	12
Ultrafiltration failure	0	0	2	7	9
Inadequate dialysis	2	2	0	0	4
Tunnel infection	0	0	1	1	2
Mechanical package	0	0	4	0	4
Patient preference			2		2
Total	3	3	15	12	33

**TABLE 4:** Risk factors for death‐censored technique survival based on the results of univariate and multivariate of Cox proportional hazards analysis

Characteristics (*n* = 204)	Univariate		Multivariate	
	HR (95%CI)	*p*	HR (95%CI)	*p*
D/P Cr 4 h				
L (<0.5)	0. 462 (0. 132–1.614)	0.226		
LA (0.5–0.64)	ref			
HA (0.65–0.80)	0.717 (0. 206–2.502)	0.602		
H (≥0.81)	2.740 (1.280–5.866)	0.009	5.700 (1.581–20.548)	0.008
Age (per year)	1.017 (0.982–1.053)	0.340		
Male Gender	0.573 (0.282–1.167)	0.125		
BMI				
<18.5 kg/m^2^	0.860 (0.274–2.703)	0.796		
18.5 ~ 23.9 kg/m^2^	ref			
24 ~ 27.9 kg/m^2^	0.689 (0.123–3.875)	0.673		
≥28 kg/m^2^	1.055 (0.338–3.294)	0.927		
DM	1.029 (0.517–2.051)	0.935		
CVD	1.289 (0.611–2.718)	0.505		
Smoking	0.538 (0.232–1.249)	0.149		
Bp <140/90 mmHg	2.357 (0.897–6.194)	0.082		
Hb (per 1 g/L)	1.002 (0.985–1.019)	0.821		
HbA_1_C (per 1 mM)	1.246 (0.991–1.568)	0.060		
Alb (per 1 g/L)	0.930 (0.871–0.993)	0.031		
SCr (per 1 μM)	1.000 (0.998–1.001)	0.539		
UA (per 1 μM)	1.001 (0.998–1.004)	0.485		
TG (per 1 mM)	0. 975 (0.710–1.338)	0.876		
TC (per 1 mM)	0. 869 (0. 633–1.194)	0.387		
cSCa (per 1 mM)	1.033 (0.583–1.830)	0.912		
P (per 1 mM)	0. 498 (0.234–1.059)	0.070		
PTH (per 1 ng/L)	0. 998 (0.995–1.000)	0.075		
cTnT (per 1 ng/ml)	0.990 (0.587–1.669)	0.969		
CRP (per 1 mg/L)	0. 994 (0.981–1.007)	0.389		
Weekly peritoneal kt/v (per 1 unit)	0. 804 (0. 300–2.157)	0.665		
Weekly residual renal kt/v (per 1 unit)	0.951 (0.502–1.802)	0.877		

Note

cSCa = serum Ca + 0.8*(4.0‐serum ALB) [if serum ALB<4g/dl].

Abbreviations: 4 h D/P Cr, dialysate:plasma creatinine ratio at 4 h; Alb, albumin; BMI, body mass index; Bp, blood pressure; CRP, C‐reactive protein; cSCa, corrected serum calcium, cTnT, cardiac troponin; CVD, cardiovascular disease; DM, diabetes mellitus; H, high; HA, high average; Hb, Haemoglobin; HbA_1_c, glycosylated haemoglobin; L, low; LA, low average; P, phosphate; PTH, parathyroid hormone; Scr, serum creatinine; TC, total cholesterol; TG, triglyceride; UA, uric acid.

### 3.3 Predictors of Baseline Peritoneal Transport Status

**FIGURE 3:**
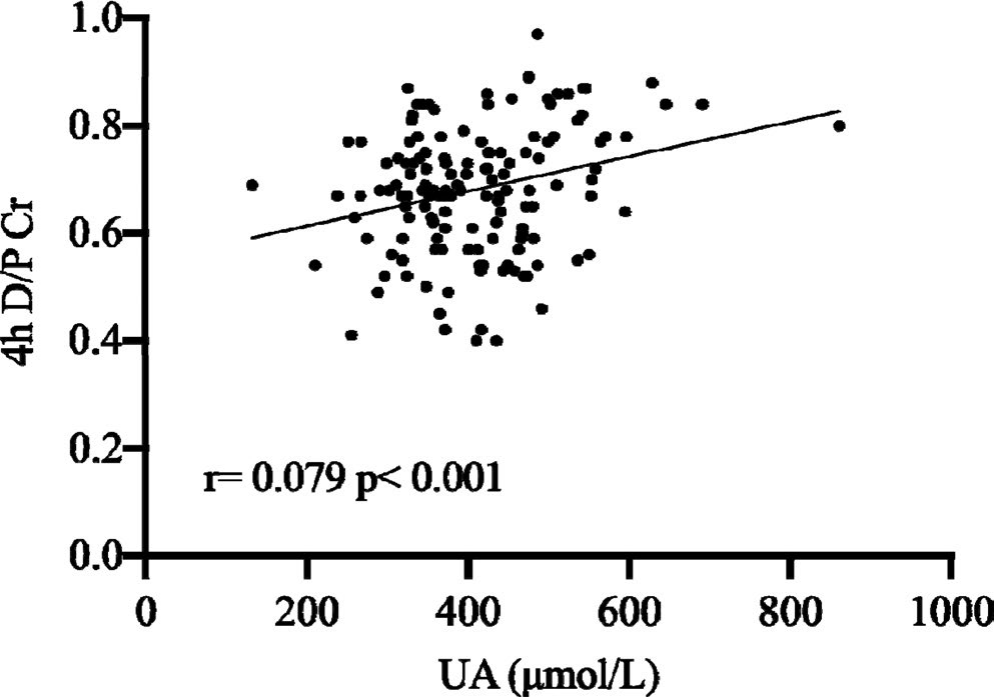
Scatter plot for correlation between serum UA concentration and 4‐h D/PCr. UA, uric acid; 4‐h D/P Cr, dialysate/plasma creatinine at 4 h

In order to explore the possible reasons for the baseline high PSTR, we performed logistic regression analysis about the clinical characteristics and found hyperuricaemia and hypoalbuminaemia were predictive of high transport status in both univariate logistic regression model (odds ratio [OR] 1.005, 95% CI 1.002 to 1.008, *p *= 0.002 and OR 0.899, 95% CI 0.830 to 0.973, *p *= 0.008, respectively) and multivariate model (adjusted OR 1.007, 95% CI 1.003 to 1.012, *p *= 0.020 and OR 0.795, 95% CI 0.678 to 0.933, *p *= 0.005, respectively) (Table 5). When peritoneal permeability was modelled as a continuous variable (4‐h D/P Cr), we got similar result. Using binary linear regression, baseline higher 4‐h D/P Cr was independently predicted by higher SUA levels (*p *< 0.001) (Figure 3). Otherwise, peritoneal permeability was not associated with the other clinical characteristics which were shown in Table 5.

**TABLE 5:** Risk Factors of Baseline High Peritoneal Transport Status in 204 Incident PD Patients based on the results of univariate and multivariate logistic regression analysis

Characteristics (*n* = 204 Events)	Univariate	Multivariate		
	OR (95%CI)	*p*	AOR (95%CI)	*p*
Age (per year)	0.982 (0.950–1.016)	0.304	0.982 (0.926–1.040)	0.531
Male Gender	1.958 (0.102–4.381)	0.102	1.474 (0.399–5.446)	0.561
BMI (per 1 kg/m^2^)	0.997 (0.897–1.115)	0.957	1.039 (0.867–1.244)	0.681
DM	0.751 (0.349–1.616)	0.464	0.967 (0.281–3.329)	0.958
CVD	0.522 (0.235–1.157)	0.110	0.770 (0.209–2.837)	0.695
Smoking	1.296 (0.486–3.456)	0.605	1.732 (0.354–8.477)	0.498
Bp <140/90 mmHg	0.533 (0. 207–1.371)	0.191	0.745 (0.148–3.744)	0.720
HbA_1_C (per 1 mM)	0.855 (0.631–1.159)	0.314	1.004 (0.659–1.532)	0.984
Hb (per 1 g/L)	0.990 (0.972– 1.009)	0.295	1.004 (0.659–1.532)	0.718
Alb (per 1 g/L)	0.899 (0.830–0.973)	0.008	0.795 (0.678–0.933)	0.005
SCr (per 1 μM)	0.999 (0.998–1.001)	0.297	1.001 (0. 999–1.003)	0.541
UA (per 1 μM)	1.005 (1.002–1.008)	0.002	1.007 (1.003–1.012)	0.020
TG (per 1 mM))	1.133 (0.813–1.579)	0.447	0.641 (0.316–1.299)	0.217
TC (per 1 mM)	0.882 (0.633–1.229)	0.459	1.816 (0.913–3.614)	0.089
cSCa (per 1 mM)	0.568 (0.273–1.179)	0.129	0.706 (0.252–1.980)	0.508
P (per 1 mM)	0.723 (0.321–1.625)	0.432	0.376 (0.078–1.813)	0.223
PTH (per 1 ng/L)	1.000 (0.999–1.002)	0.509	1.000 (0.998–1.002)	0.771
cTnT (per 1 ng/ml)	0.643 (0.279–1.479)	0.298	0.412 (0.113–1.506)	0.180
CRP (per 1 mg/L)	0.982 (0. 953–1.011)	0.218	0.977 (0.939–1.018)	0.267
Weekly Renal kt/v (per 1 unit)	0.747 (0. 356–1.568)	0.440	1.005(0.307–3.287)	0.993

Note

cSCa = serum Ca + 0.8*(4.0‐serum ALB) [if serum ALB<4g/dl].

Abbreviations: 4 h D/P Cr, dialysate:plasma creatinine ratio at 4 h; Alb, albumin; BMI, body mass index; Bp, blood pressure; CRP, C‐reactive protein; cSCa, corrected serum calcium, cTnT, cardiac troponin; CVD, cardiovascular disease; DM, diabetes mellitus; Hb, haemoglobin; H, high; HA, high average; HbA_1_c, glycosylated haemoglobin; L, low; LA, low average; P, phosphate; PTH, parathyroid hormone; Scr, serum creatinine; TC, total cholesterol; TG, triglyceride; UA, uric acid.

### 3.4 Uric acid induces EMT of cultured human peritoneal mesothelial cells in a dose‐dependent manner

**FIGURE 4:**
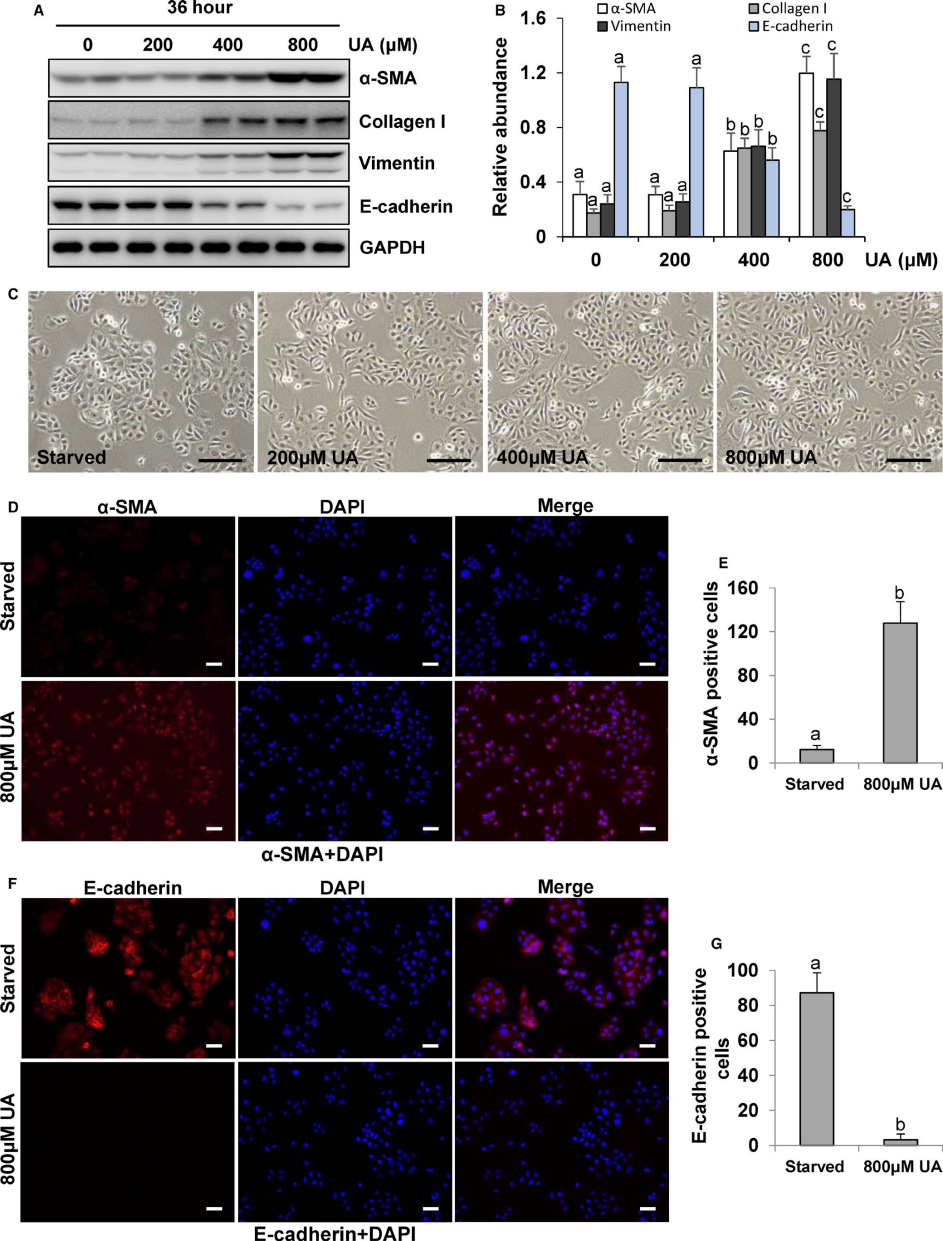
Uric acid induces EMT of cultured human peritoneal mesothelial cells in a dose‐dependent manner. (A) Cell lysates were subjected to immunoblot analysis using antibodies to α‐SMA, collagen I, vimentin, E‐cadherin, or GAPDH. (B) Expression levels of α‐SMA, collagen I, vimentin and E‐cadherin were quantitated by densitometry and normalized with GAPDH. (C) Photomicrographs illustrated the characterization of the HPMCs phenotype. (D) Photomicrographs illustrating immunofluorescence co‐staining of α‐SMA and DAPI. (E) The positive area of α‐SMA was quantitatively analysed. (F) Photomicrographs illustrating immunofluorescence co‐staining of E‐cadherin and DAPI. (G) The positive area of E‐cadherin was quantitatively analysed. Data are represented as the means ± SD. Bars with different letters (a–c) for each molecule are significantly different from one another (*p *< 0.05). Scale bars in c = 500 μm, d = 50 μm and f = 50 μm

The present results noted us that baseline uric acid levels were obviously associated with both all‐cause death and high PSTR, but how SUA effects on peritoneal structure and function is still unknown. Therefore, we performed in vitro experiments on human peritoneal mesothelial cells (HPMCs) with uric acid. EMT of peritoneal mesothelial cells occurred after peritoneal injury and involved in the progression of peritoneal fibrosis.^34^ As the first step towards understanding whether uric acid is participated in peritoneal fibrosis, we examined the expression of EMT markers in the cultured HPMCs stimulated with uric acid at 36 h. Exposure of HPMCs to uric acid substantially reduced the protein levels of E‐cadherin, an epithelial cell marker, and increased expression of α‐smooth muscle actin (α‐SMA), collagen I and vimentin, three mesenchymal markers, which occurred in a dose‐dependent manner, with a maximum effect at 800 μM (Figure 4A,B). Moreover, uric acid was found to dose dependently induce the morphological transition of HPMCs, which stimulated HPMCs to lose their cobblestone appearance and to become spindle‐like, thus resembling mesenchymal cell morphology (Figure 4C). In addition, immunofluorescence assay showed that the expression of α‐SMA was significantly increased in response to 800 μM uric acid (Figure 4D,E); however, the level of E‐cadherin expression was in the basal state and that its expression was decreased stimulated by 800 μM uric acid (Figure 4F,G). Collectively, these results indicated that uric acid can induce EMT in peritoneal mesothelial cells in a dose‐dependent manner.

Further results indicated that uric acid had the additive effect of EMT with 2.5% HG peritoneal dialysis fluid. Expression levels of α‐SMA, collagen I and vimentin were higher in combined use of UA and HG than separate use (Supplemental Figure S1). Moreover, 800 μM uric acid could increase ROS production according to the DCFH‐DA immunofluorescence (Supplemental Figure S2), suggesting that UA contributed to oxidative stress in HPMCs.

### 3.5 Uric acid induces EMT of cultured human peritoneal mesothelial cells in a time‐dependent manner

**FIGURE 5:**
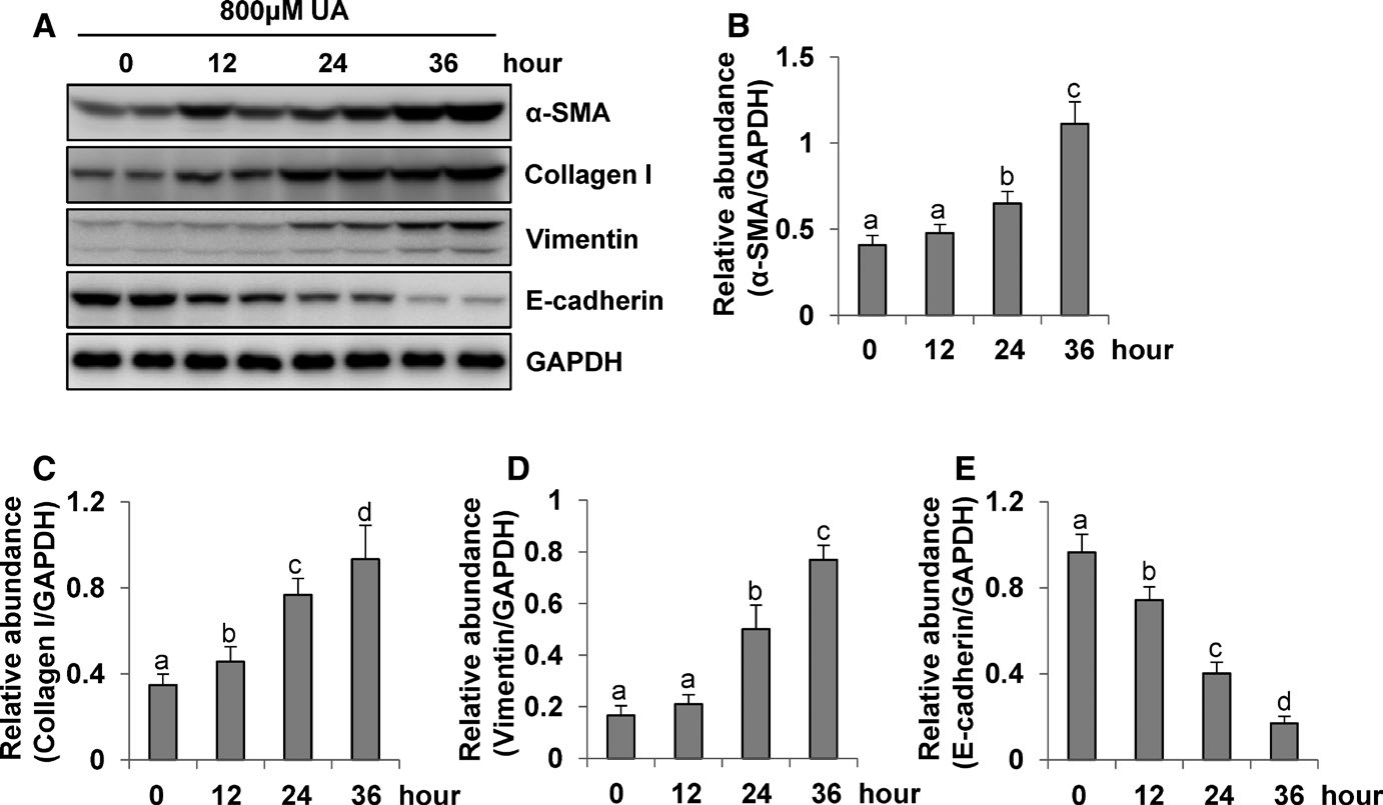
Uric acid induces EMT of cultured human peritoneal mesothelial cells in a time‐dependent manner. (A) Cell lysates were subjected to immunoblot analysis using antibodies to α‐SMA, collagen I, vimentin, E‐cadherin or GAPDH. (B–E) Expression levels of α‐SMA, collagen I, vimentin and E‐cadherin were quantitated by densitometry and normalized with GAPDH. Data are represented as the means ± SD. Bars with different letters (a–c) for each molecule are significantly different from one another (*p *< 0.05)

Moreover, we also explored the level of EMT marker expression in the HPMCs stimulated with uric acid at 800 μM in different periods of time. As shown in Figure 5, the α‐SMA, collagen I and vimentin were barely detected in the HPMCs of 0 h, but their expression was increased at 12 and 24 h after uric acid exposure, and further elevated at 36 h (Figure 5A–D). E‐cadherin expression was clearly observed in the HPMCs at 0 h, gradually decreasing between hours 12 and 24, and further reduced at 36 h (Figure 5A,E). Taken together, these results illustrated that uric acid induced EMT in peritoneal mesothelial cells in a time‐dependent manner.

### 3.6 Uric acid induces EMT by the activation of the TGF‐β1/Smad3 signalling pathway and nuclear transcription factors in peritoneal mesothelial cells

**FIGURE 6:**
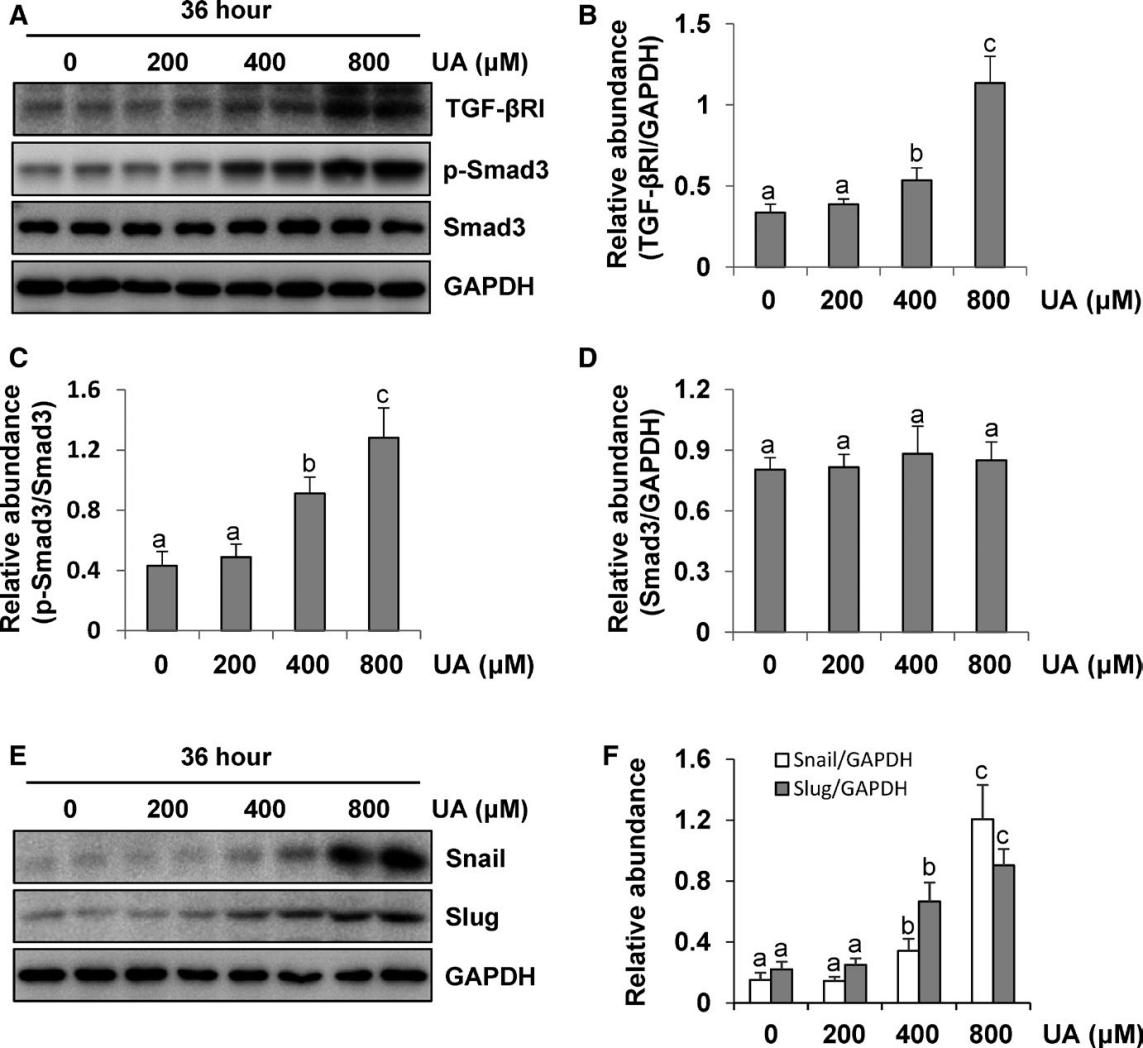
Uric acid induces EMT by activation of the TGF‐β1/Smad3 signalling pathway and nuclear transcription factors in peritoneal mesothelial cells. (A) Cell lysates were subjected to immunoblot analysis using antibodies to TGF‐βRI, p‐Smad3, Smad3 or GAPDH. (B and D) Expression levels of TGF‐βRI and Smad3 were quantitated by densitometry and normalized with GAPDH. (C) Expression level of p‐Smad3 was quantified by densitometry and normalized with total Smad3. (E) Cell lysates were subjected to immunoblot analysis using antibodies to Snail, Slug or GAPDH. (F) Expression levels of Snail and Slug were quantitated by densitometry and normalized with GAPDH. Data are represented as the means ± SD. Bars with different letters (a–c) for each molecule are significantly different from one another (*p *< 0.05)

It is well known that the TGF‐β1/Smad3 signalling pathway plays an important role for the development of EMT in peritoneal mesothelial cells.^34^ We thus examined whether uric acid would regulate EMT by the activation of this signalling pathway in HPMCs. As shown in Figure 6A–D, exposure to uric acid at 36 h dose dependently increased the expression of TGF‐βRI and phosphorylation of Smad3 in HPMCs, but had no impact on the expression of total Smad3. As for Snail and Slug are two critical nuclear transcription factors that mediate the EMT through suppressing transcription of E‐cadherin and other epithelial markers,^35^ we also examined the impact of uric acid on their expression. Elevated expression of Snail and Slug in a dose‐dependent manner was observed in the HPMC exposure to uric acid at 36 h (Figure 6E,F). Collectively, these data demonstrated that uric acid induces EMT by mechanisms associated with the TGF‐β1/Smad3 signalling pathway and nuclear transcription factors in peritoneal mesothelial cells.

### 3.7 Uric acid facilitates the proliferation and migration of peritoneal mesothelial cells

**FIGURE 7:**
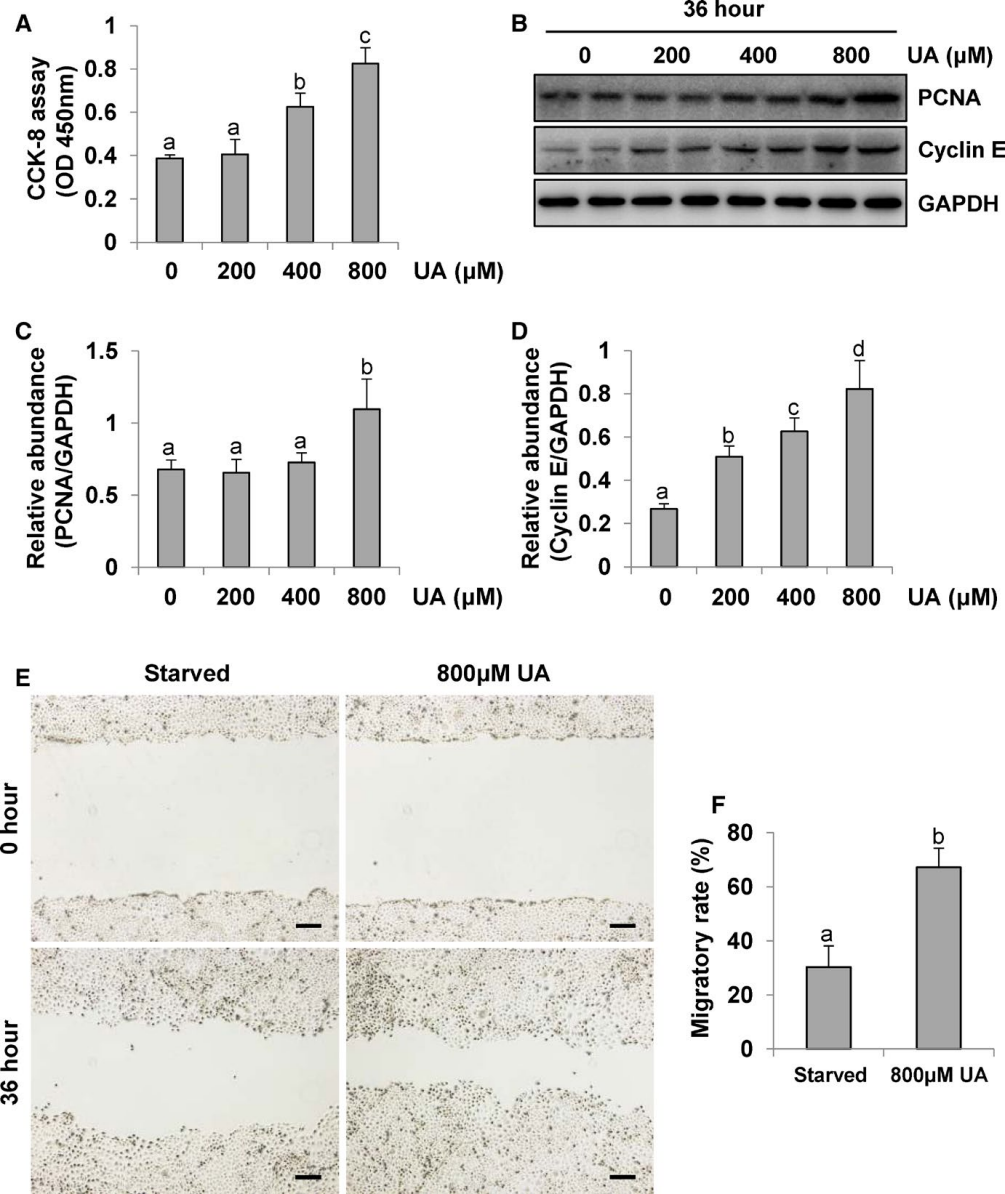
Uric acid facilitates the proliferation and migration of peritoneal mesothelial cells. (A) Results from the CCK‐8 assays showing the dose‐dependent cell proliferation induced by uric acid in the HPMCs. (B) Cell lysates were subjected to immunoblot analysis using antibodies to PCNA, Cyclin E or GAPDH. (C and D) Expression levels of PCNA and Cyclin E were quantitated by densitometry and normalized with GAPDH. (E and F) The wound‐healing assay and quantitative analysis results demonstrated that uric acid led to a significant increase in the migration ability of HPMCs at 36 h. Data are represented as the means ± SD. Bars with different letters (a–c) for each molecule are significantly different from one another (*p *< 0.05). Scale bars in e = 500 μm

To investigate whether uric acid is also involved in proliferation of HPMCs, a CCK‐8 assay was performed. The detection revealed a dose‐dependent gradual increase in cell proliferation with a higher uric acid concentration (Figure 7A). Cell proliferation includes four different stages of the cell cycle (G0/G1, S, G2 and M),^36^ since proliferating cell nuclear antigen (PCNA) affects DNA synthesis in the S phase of cells and cyclin E affects the process from the G1 phase to the S phase, and we found that the expression of PCNA and cyclin E in HPMCs exposure to uric acid at 36 h was increased in dose‐dependent manner (Figure 7B–D). In addition, to test whether uric acid involves in the migration potential of HPMCs, the wound‐healing assay was performed. Wound‐healing assay demonstrated that uric acid led to a significant increase in the migration ability of HPMCs at 36 h (Figure 7E,F). Taken together, these data suggested that uric acid promotes the proliferation and migration of peritoneal mesothelial cells.

### 3.8 The uric acid concentration in dialysate

Finally, in order to clarify the difference between uric acid concentration in PD solution and serum, forty‐three PD effluent samples were collected after 4 h dwelling in the abdominal cavity when performed the first PET test. We measured the concentration of uric acid in PD effluent and found that the concentration of uric acid was lower in PD effluent compared with that in serum (*p *< 0.001). Compared with patients in low average group, the concentration of uric acid in PD effluent was higher in high transporters (Supplemental Figure S3).

## 4 DISCUSSION

This study retrospectively evaluated a cohort of patients that had their initial peritoneal membrane permeability analysed as categorical variable (H, HA, LA and L transport status). In our research, transport classes were significantly associated with death‐censored technique survival. Compared with LA group, baseline H transporters have a more than 5 times higher risk of technical failure (*p *= 0.008). SUA was significantly associated with baseline high PSTR (*p *= 0.020) and independently predicted mortality (*p *< 0.001). Furthermore, total cholesterol (TC) was inversely associated with mortality (*p *= 0.018) and albumin was significantly associated with baseline high PSTR (*p *= 0.005). Subsequent in vitro experiments suggested that UA may cause high baseline PSTR through the way of inducing EMT of HPMC by activating the TGF‐β1/Smad3 signalling pathway and nuclear transcription factors mechanistically.

Since the famous Canada‐USA prospective cohort study proved that high solute transporters are associated with a greater risk of either technique failure or mortality for patients who received CAPD,^12^ including the largest investigation from Australian and New Zealand Dialysis and Transplant Association (ANZDATA) Registry,^13^ more and more studies had confirmed that the high transport of peritoneal solutes is one of the risk factors for poor prognosis.^37^ Our findings were broadly consistent with those of previous studies. The suggested mechanism of adverse outcomes was mainly concentrated on peritoneal ultrafiltration failure and fluid overload do to rapid glucose absorption followed by reduction in osmotic gradient and abundant loss of protein in the dialysate leading to malnutrition.^38^, ^39^, ^40^ It was apparently to see in the present study that high transporter was significantly related to low albuminaemia and ultrafiltration insufficiency was a primary cause of dropout from PD, and H transport group accounted for 77.8% of patients who switched to HD due to ultrafiltration failure. Another vital aspect of high PSTR is that more and faster glucose absorption will bring on disadvantages such as insulin resistance, dyslipidaemia and malnutrition.^41^ Moreover, glucose degradation products (GDPs) generated from dextrose dialysate may serve as stimuli for peritoneum injuries and predispose patients to develop peritonitis, and these processes may cause high PSTR in turn. In our findings, peritonitis was a primary reason leading to technical failure.

However, there were also some other researches demonstrated different conclusions that high PSTR may not play critical roles in technical failure or death.^42^, ^43^ Well to known, high PSTR preferred to more frequent cycles with shorten dwell time rather than CAPD. In recent years, after the introduction of automated peritoneal dialysis (APD), it has been considered that the fluid overload can be alleviated while fluid balance can be achieved due to the faster fluid removal. In this way, APD may improve the clinical outcomes among patients with high PSTR.^44^ In the publication from the ANZDATA Registry, poor prognosis was associated with fast transporters only on CAPD but not on APD.^13^ Another larger study of 4128 patients showed that APD leads to better survival of fast transporters.^45^ On the other hand, with the utilization of biocompatible neutral pH, low GDP dialysate, peritoneal ultrafiltration may be increased and the function of peritoneum can also be preserved.^11^, ^46^ For patients who fail to achieve ultrafiltration goals with CAPD, APD implementation in combination with icodextrin dialysate could prolong technique survival.^47^

In our present study, even after adjustment for nutrition and inflammation factors such as serum albumin, CRP, concentrations of creatinine, phosphorus and body mass index, hyperuricemia was still an independent predictor of all‐cause mortality. To our knowledge, UA is the metabolic end product of nucleic acid purine which is mainly eliminated via renal excretion. Hyperuricaemia is very common in CKD patients due to the decreased ability of the kidneys to remove uric acid. In individuals at high risk for cardiovascular disease, despite uric acid has been proven to be a potent radical scavenger and antioxidant,^48^ the concentration of SUA is still an independent predictor of mortality.^49^, ^50^ ESRD patients are obviously at high risk of cardiovascular disease, but in a recent published review, the relationship between serum concentrations of UA and mortality was discordant in five studies of patients with ESRD receiving PD.^51^ Our result was consistent with Feng et al.^52^ and Xia et al.^53^ which concluded that high serum UA levels were associated with higher mortality. While a prospective cohort study from South Korea came to an opposite conclusion that a lower time‐averaged SUA level <5.5 mg/dl predicted all‐cause mortality in patients with PD account for a reduced total antioxidant capacity.^54^ The other two studies from China also showed no association between hyperuricaemia and mortality.^55^, ^56^ The discordance may be a consequence of differences in sample size which from hundreds to thousands of participates, length of follow‐up, use of drugs for controlling SUA, biochemical and clinical index and analysis of risk variables for cardiovascular disease. Experimental and clinical research showed that intracellular UA exerts prooxidant effects^57^ while extracellular UA exerts antioxidant effects.^58^ Under normal circumstances, intracellular concentrations of UA are lower than extracellular despite intracellular UA production.^59^ Some scholars believed that acid‐base status can affect the rate of UA transport between intracellular and extracellular compartments. Patients with ESRD may confer lower intracellular concentrations of UA by enhancing intracellular to extracellular efflux of UA. Continuous peritoneal dialysis makes the serum bicarbonate concentration higher than that of intermittent haemodialysis patients, resulting in intracellular UA levels may be higher in PD patients compared to those receiving haemodialysis.^51^ The prooxidant effects within endothelial cells or vascular smooth muscle cells are inseparable from cardiovascular disease. Accumulated evidence indicated that oxidative stress (OS) is increased in PD patients, causing fibrosis of the peritoneum and leading to loss of ultrafiltration capacity ultimately.^60^ Since UA was proved to contribute to oxidative stress in HPMCs in the vitro experiment, it can be inferred that uric acid may cause changes in peritoneal transport characteristics through oxidative stress damage.

On the other hand, the higher levels of serum UA were also believed to be a marker of better nutrition in patients with dialysis.^61^ The DOPPS study (a large ongoing international prospective cohort study of 4637 haemodialysis patients [median follow‐up, 23 months]) concluded that baseline serum uric acid level was reversely and independently related to all‐cause and CV mortality, and it was explained in part that higher levels of serum UA were a marker for better nutritional status.^62^ As for peritoneal dialysis, several studies revealed a ‘U‐shaped’ relationship between UA levels and all‐cause mortality.^63^, ^64^ Lower serum UA levels had been identified as reflecting deteriorated intake of nutrition including protein and calorie. Even so, we had not reached a similar conclusion about SUA, as one of the nutrition indicators and traditional CVD risk factors as well, and we found an inverse relationship between total cholesterol and all‐cause mortality. Malnutrition‐related hypocholesterolaemia often increases mortality in patients received dialysis.^63^, ^65^ Arsalan et al. suggested PD patients with TC levels ≤125 mg/dl (3.24 mM) had a statistically significant increased risk of an all‐cause mortality.^65^ Recently, a large cohort study of 8032 PD patients from China Taiwan also concluded higher level of TC (>200 mg/dl) in both first and third years of dialysis had significantly lower risk of mortality.^66^ Although our finding was consistent with several previous study, the role of dyslipidaemia on mortality in subjects receiving dialysis is quite complicated.

Since SUA was an independently predictor of mortality and baseline high PSTR in our study, we performed cytology experiments to investigate how SUA effects on peritoneal structure and function, especially try to explain the relationship between uric acid and high PSTR. According to our results, UA promotes the EMT process of HPMCs, and uric acid had the additive effect of EMT with high glucose peritoneal dialysis fluid. During the progression of EMT, MCs acquire the ability to synthesize large amounts of extracellular matrix components (ECM) and express high levels of cytokines related to inflammation, fibrotic and angiogenic processes.^29^, ^67^ Among these cytokines, VEGF played a key role in peritoneal neoangiogenesis and high PSTR. Aroeira et al. demonstrated that MCs undergone EMT were the main source of VEGF in PD patients and related to high peritoneal transport rate.^68^ Do et al. also found dialysate VEGF revealed a significant association with EMT of PMC cultured from overnight effluent when adjusted by effluent CA125.^69^ As mentioned above, the TGF‐β1/Smad signalling pathway played a pivotal role in EMT.^27^, ^34^ Just recently, Duan et al. reported that TGF‐β1/Smad3 pathway and mitogen‐activated protein kinase (MAPK)/P38 pathway involved in the effects of UA‐induced EMT of HPMCs.^70^ Our present study also investigated the signalling cascades involved in the effects of UA‐induced EMT. Our cell experiment confirmed that TGF‐β1/Smad3 pathway is regulated by UA, which can directly promote EMT. Meanwhile, the exposure of peritoneal mesothelial cells to UA elevated expression of Snail and Slug in a dose‐dependent manner, which suggested that UA‐induced EMT was closely associated with the activation of nuclear transcription factors. In combination with the results above, we deduced that UA can promote the EMT process of peritoneal mesothelial cells, which may be closely related to baseline high PSTR.

There are some limitations in this study. Firstly, we only collected baseline clinical data of PD patients, and relationships may be neglected if some biochemical indexes vary from time to time thus needed be analysed as time‐dependent variable. Secondly, diet or pharmacological treatment both be neglected in our research, which may have influence on data analysis and affect us predicting long‐term outcomes of patients. Thirdly, we excluded a part of patients due to unavailability of baseline PET measurements, which may carry a risk for bias. Moreover, our data only focused on clinical examinations and basic personal information instead of providing information including environmental conditions during exchanging dialysates, personal finance circumstances in affording PD treatment and social support of patients, which can all affect the clinical outcomes of patients. Finally, although we enrolled patients from diverse PD centres, our samples are rather small, a larger scale cohort study with longitudinal follow‐up is needed to further confirm our findings. However, our studies have its strengths in providing comprehensive insight into the mechanism of UA‐caused EMT and high PSTR. To our knowledge, this is the first study to investigate relationship between hyperuricaemia, high PSTR and related pathological changes in peritoneum.

In conclusion, the present study demonstrated that baseline high PSTR was significantly associated with death‐censored technique survival. Baseline higher levels of UA were the significant independent predictors of all‐cause mortality. Hyperuricaemia and hypoalbuminaemia served as risk factors in relation to baseline high PSTR. TC may be a protective factors for prognosis of PD. Mechanically, UA can promote the EMT process of peritoneal mesothelial cells in *vitro*, which may be closely related to baseline high PSTR and was an important reason of poor outcomes in CAPD patients. More attentions should be paid to PD patients with hyperuricaemia. Further studies are warranted to confirm the findings of our study.

## CONFLICT OF INTEREST

No conflicts of interest, financial or otherwise, are declared by the author(s).

## AUTHOR CONTRIBUTIONS

**Guansen Huang:** Data curation (equal); Formal analysis (equal); Investigation (equal); Methodology (equal); Software (equal); Writing‐original draft (equal). **Yi Wang:** Data curation (equal); Formal analysis (equal); Investigation (equal); Methodology (equal); Software (equal); Writing‐original draft (equal). **Yingfeng Shi:** Data curation (equal); Formal analysis (equal); Investigation (equal); Methodology (equal); Software (equal); Writing‐original draft (equal). **Xiaoyan Ma:** Investigation (equal); Methodology (equal). **Min Tao:** Investigation (equal); Methodology (equal). **Xiujuan Zang:** Funding acquisition (equal); Investigation (equal); Methodology (equal). **Ying‐Hui Qi:** Investigation (equal); Methodology (equal). **Cheng Qiao:** Investigation (equal); Methodology (equal). **Lin Du:** Investigation (equal); Methodology (equal). **Lili Sheng:** Funding acquisition (equal); Investigation (equal); Methodology (equal). **Shougang Zhuang:** Funding acquisition (equal); Writing‐original draft (equal); Writing‐review & editing (equal). **Na Liu:** Conceptualization (equal); Data curation (equal); Formal analysis (equal); Funding acquisition (equal); Writing‐original draft (equal); Writing‐review & editing (equal).

## Supporting information

Fig S1

Fig S2

Fig S3

## Data Availability

The data that support the findings of this study are available from the corresponding author upon reasonable request.
