# Supplementary figures and images for: The prognosis and risk factors of baseline high peritoneal transporters on patients with peritoneal dialysis
Source: J Cell Mol Med. 2021 Jul 26;25(18):8628–44. doi: 10.1111/jcmm.16819 (PMC8435427; doi:10.1111/jcmm.16819)

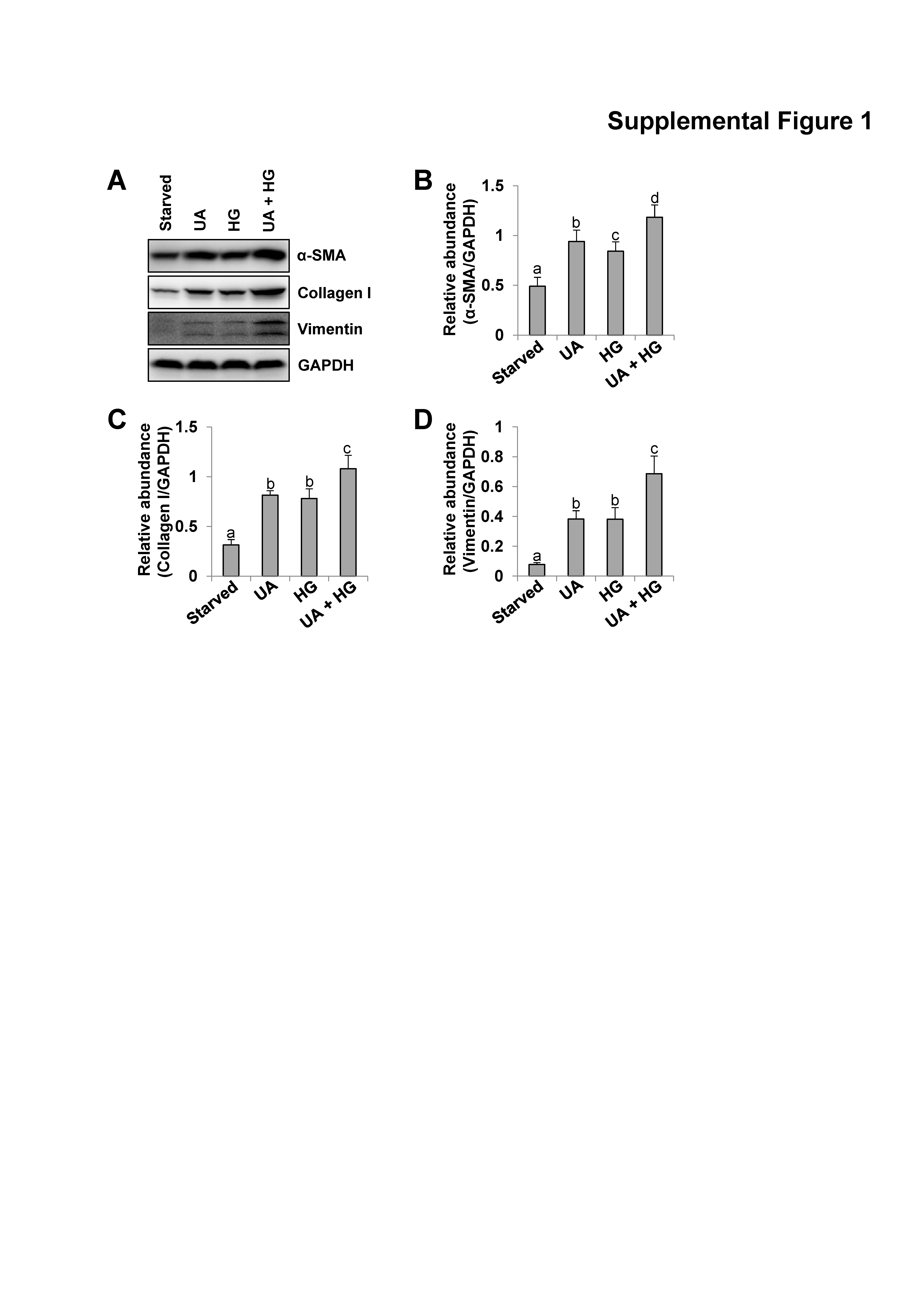

Supplement: Supplementary file 1 — Fig S1 [file JCMM-25-8628-s002.tiff]

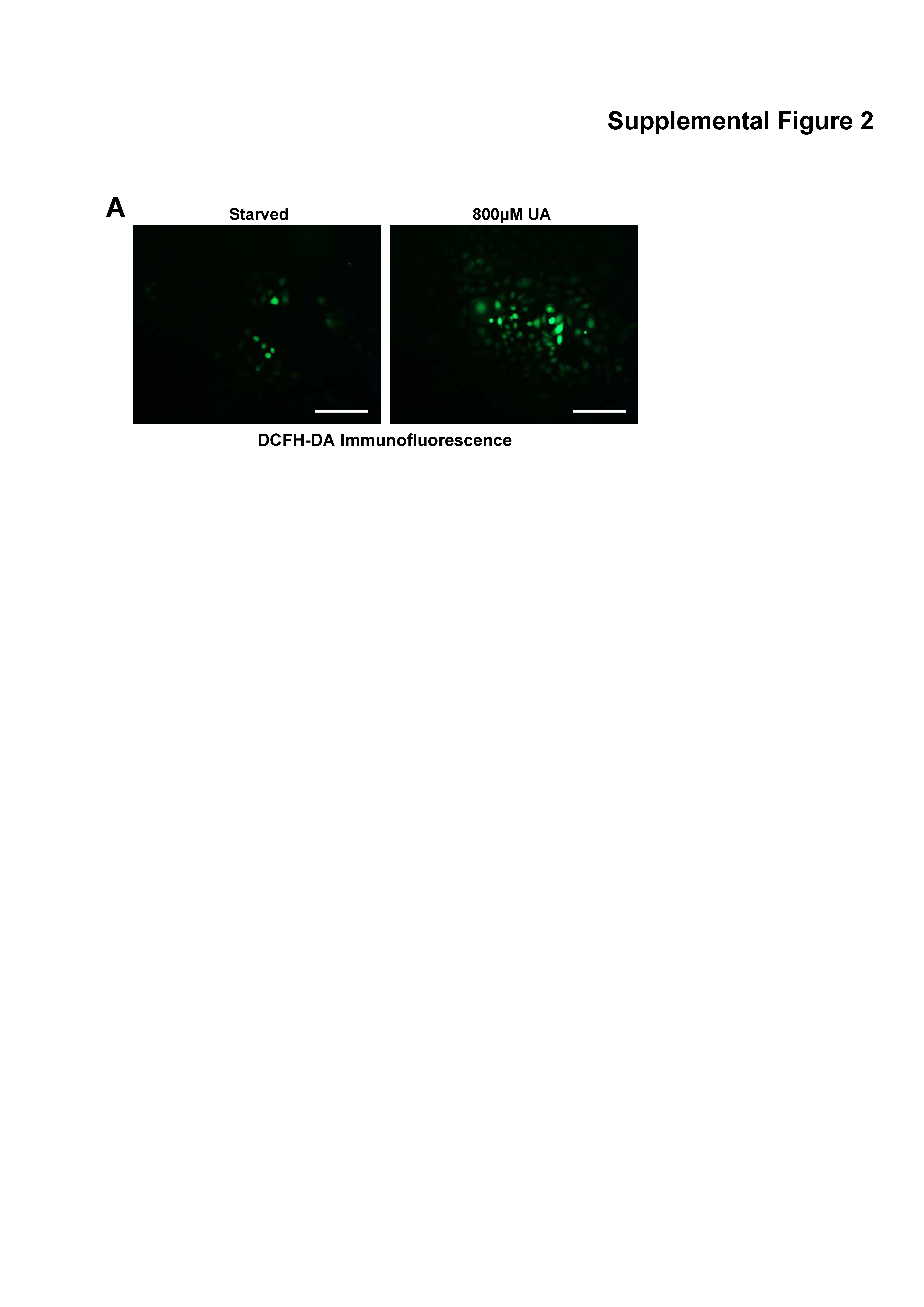

Supplement: Supplementary file 2 — Fig S2 [file JCMM-25-8628-s003.tiff]

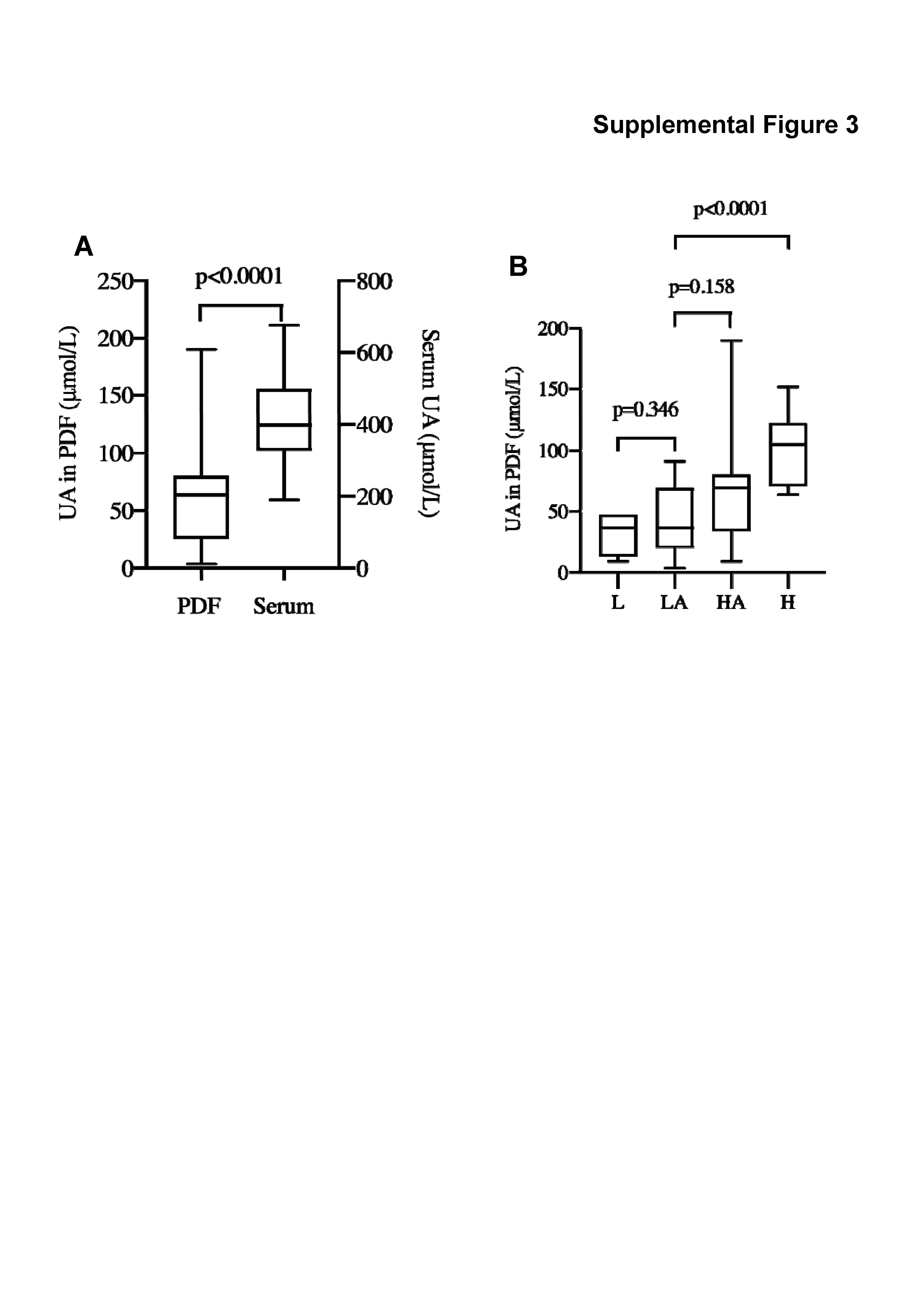

Supplement: Supplementary file 3 — Fig S3 [file JCMM-25-8628-s001.tiff]
